# Early-Onset Neonatal Sepsis Caused by *Globicatella sanguinis/sulfidifaciens* : Case Report

**DOI:** 10.1055/a-2913-6821

**Published:** 2026-07-31

**Authors:** Fredrick Odhiambo Otieno, Rafael Fonseca, Lemuel Aigbibvalu

**Affiliations:** 1 Division of Pediatric Hospital MedicineDepartment of Pediatrics 12338 The University of Texas Medical Branch at GalvestonGalvestonTexasUnited States; 2 Division of NeonatologyDepartment of Pediatrics 12338 The University of Texas Medical Branch at GalvestonGalvestonTexasUnited States

**Keywords:** neonatal sepsis, *Globicatella sanguinis*, bacteremia, early-onset sepsis, cord avulsion, rare pathogens, CLSI breakpoints

## Abstract

**Background***Globicatella sanguinis* is a rare α-hemolytic gram-positive coccus increasingly recognized as a human pathogen but remains an uncommon cause of neonatal invasive infection. Reported neonatal cases are limited, and optimal antimicrobial therapy is uncertain because Clinical and Laboratory Standards Institute (CLSI) susceptibility breakpoints are unavailable.

**Case Presentation** A term female neonate born at 39 ^3/7^ weeks via vacuum-assisted vaginal delivery after an in vitro fertilization pregnancy developed respiratory distress and lethargy shortly after birth. Delivery was complicated by umbilical cord avulsion, causing acute blood loss and hypovolemia. Initial evaluation demonstrated metabolic acidosis, leukocytosis, bandemia, and a rapid hematocrit decline requiring packed red blood cell transfusion. Blood culture obtained at birth grew *Globicatella sanguinis/sulfidifaciens* within 12 hours, whereas cerebrospinal fluid culture remained sterile. Empiric ampicillin and gentamicin were initiated; repeat blood cultures were negative. Susceptibility testing yielded minimum inhibitory concentration values without CLSI interpretation. Following infectious disease consultation, the infant completed a 10-day course of intravenous ampicillin from the first negative blood culture and recovered without complications.

**Conclusion** This case highlights the diagnostic overlap between hemorrhagic shock and early-onset neonatal sepsis and emphasizes the management challenges posed by rare pathogens lacking standardized susceptibility criteria and evidence-based neonatal treatment recommendations.

## Introduction

Early-onset neonatal sepsis (EOS) remains a significant cause of neonatal morbidity and mortality, with Group B *Streptococcus* and *Escherichia coli* accounting for 66% of all cases. 1 However, advances in diagnostic microbiology, including matrix-assisted laser desorption ionization time-of-flight mass spectrometry (MALDI-TOF MS), 16S ribosomal RNA (rRNA) gene sequencing, and sodA polymerase chain reaction (PCR), have facilitated identification of uncommon organisms as clinically significant causes of neonatal infection. 2

*Globicatella sanguinis* is an α-hemolytic, catalase-negative, gram-positive coccus that may be misidentified as viridans streptococci or enterococci due to overlapping phenotypic characteristics. 3 Human infections are rare and have been reported primarily in adults, with bacteremia, urinary tract infections, meningitis, and osteoarticular infections. 3 Neonatal infections due to *Globicatella* species remain exceedingly uncommon, with only isolated cases reported without standardized treatment guidelines to inform management. 4 Furthermore, no Clinical and Laboratory Standards Institute (CLSI) breakpoints exist for susceptibility interpretation of this organism, introducing significant therapeutic uncertainty when infection is identified.

This report describes a term neonate with early-onset bacteremia due to *Globicatella sanguinis/sulfidifaciens* identified during evaluation for respiratory distress and hemodynamic instability following delivery complicated by cord avulsion and acute blood loss.

## Case Presentation

A female infant was born at 39 ^3/7^ weeks’ gestation to a 29-year-old gravida 1 para 1 mother following an uncomplicated in vitro fertilization pregnancy. Prenatal care was appropriate, and pregnancy dating was confirmed by first-trimester ultrasonography. Mother had no history of diabetes mellitus, HIV infection, organ transplantation, immunosuppressive therapy, autoimmune disease, malignancy, chronic kidney or liver disease, or other conditions associated with immunocompromise. There was no history of recurrent urinary tract infections, invasive procedures during pregnancy. Artificial rupture of membranes occurred approximately 15 h before delivery. Maternal Group B *Streptococcus* was negative. No known maternal animal exposure during the gestational period.

The infant was delivered vaginally with vacuum assistance. Delivery was complicated by umbilical cord avulsion, resulting in suspected acute blood loss and hypovolemia. Apgar scores were 6 and 7 at 1 and 5 minutes, respectively. Initial resuscitation included drying, stimulation, and deep suctioning.

Birth weight was 3,440 g (appropriate for gestational age), length 55 cm, and head circumference 34 cm. Shortly after birth, the infant developed respiratory distress characterized by intermittent grunting and required neonatal intensive care unit admission on continuous positive airway pressure (CPAP) at 5 cm H _2_ O with an FiO _2_ of 0.30.

On admission, the infant appeared pale, mottled, lethargic, and hypotonic. Vital signs ranges were as follows: temperature, 97.8°F (36.6°C) to 99.1°F (37.3°C); heart rate, 175 to 178; respiratory rate, 28 to 32; blood pressure 44 to 62/24 to 26 mm Hg with mean arterial pressures of 27 to 32; oxygen saturation 94 to 96% on CPAP. Physical examination was notable for mild respiratory distress with intermittent grunting, molding, and caput succedaneum. Pulses were palpable, and no cardiac murmur was appreciated. Neurological examination demonstrated generalized hypotonia with preserved Moro and suck reflexes.

Arterial blood gas analysis showed metabolic acidosis (7.22/30/129/12/−14.2). The infant received a 10 mL/kg normal saline bolus for hypovolemia and hypotension. Empiric ampicillin and gentamicin were initiated following sepsis evaluation.

A chest radiograph obtained demonstrated subtle bilateral perihilar interstitial opacities consistent with transient tachypnea of the newborn. The infant was maintained nil per os with intravenous dextrose-containing fluids (Dextrose 10%) at 60 mL/kg.

### Hospital Course

#### Infectious Disease Workup

Initial laboratory evaluation demonstrated a white blood cell count of 31.35 × 10 ^3^ /μL (reference range, 9.10–34.00 × 10 ^3^ /μL), red blood cell count of 3.42 × 10 ^6^ /μL (4.10–6.70 × 10 ^6^ /μL), hemoglobin concentration of 12.0 g/dL (15.0–22.0 g/dL), and platelet count of 250 × 10 ^3^ /μL (135–3,610 ^3^ /μL). Differential leukocyte count revealed 61% segmented neutrophils, 14% bands (reference range, 0–8%), and 3% metamyelocytes, consistent with bandemia and a left shift. The immature-to-total neutrophil (I/T) ratio was elevated at 0.52 (normal <0.20). Blood cultures obtained at admission became positive within 12 hours, yielding gram-positive cocci that were subsequently identified as *G. sanguinis/G. sulfidifaciens* . Cerebrospinal fluid cultures remained sterile. Repeat blood cultures obtained at 48 h of life demonstrated no growth, indicating rapid clearance of bacteremia following initiation of antimicrobial therapy.

Antimicrobial susceptibility testing of the *G. sanguinis/G. sulfidifaciens* isolate was performed at the ARUP laboratory, and the resulting minimum inhibitory concentration (MIC) values were as presented in Table 1 .

**Table 1:** ARUP susceptibility results for *Globicatella sanguinis* / *sulfidifaciens* .

ARUP susceptibility results		
Antimicrobial agent	MIC (μg/mL)	Interpretation
Ceftriaxone	≥4	No interpretation (no CLSI breakpoints)
Meropenem	1	No interpretation (no CLSI breakpoints)
Penicillin	0.25	No interpretation (no CLSI breakpoints)
Vancomycin	0.25	No interpretation (no CLSI breakpoints)

Abbreviations: CLSI, Clinical and Laboratory Standards Institute; MIC, minimum inhibitory concentration.

Note: Interpretive information—at the present time, there are no CLSI guidelines for performance and/or interpretation of susceptibility testing for the above organism and/or indicated antimicrobial agent(s). Interpretive results: Interpretive information—susceptibility not specified.

#### Hematologic and Hemodynamic Course

During the first 24 hours of life, the infant developed progressive anemia, with hematocrit declining from 38% at birth to 25.8%. Repeat complete blood count demonstrated a white blood cell count of 24.87 × 10 ^3^ /μL (reference range, 9.10–34.00 × 10 ^3^ /μL), red blood cell count of 2.62 × 10 ^6^ /μL (4.10–6.70 × 10 ^6^ /μL), hemoglobin concentration of 12.0 g/dL (15.0–22.0 g/dL), hematocrit of 25.8% (44.0–70.0%), and platelet count of 212 × 10 ^3^ /μL (135–3,610 ^3^ /μL). Differential leukocyte count revealed 28% segmented neutrophils and 7% band forms, with an I/T ratio of 0.20. The anemia was attributed to acute blood loss secondary to umbilical cord avulsion at delivery. On day 2 of life, the infant received a packed red blood cell transfusion (20 mL/kg), resulting in hematologic improvement, with posttransfusion hemoglobin and hematocrit increasing to 15.0 g/dL and 44%, respectively. Cranial ultrasound and abdominal ultrasound findings were unremarkable.

#### Antimicrobial Management

Pediatric infectious disease consultation was obtained on day of life (DOL) 3. Given clinical improvement, sterile cerebrospinal fluid cultures, and negative repeat blood cultures, gentamicin was discontinued. Ampicillin monotherapy continued for a total of 10 days from the first negative blood culture.

#### Outcome

During hospitalization, the infant experienced a single apnea and bradycardia episode on DOL 2, requiring moderate stimulation but otherwise demonstrated progressive clinical improvement. Respiratory distress resolved within 24 hours from birth; enteral feeding was advanced without difficulty. She completed full antibiotic course and was discharged home without complications.

## Discussion

*Globicatella sanguinis* was first recognized as a human pathogen in the 1990s. 2 A close phylogenetic relationship exists between *G. sanguinis* and *G. sulfidifaciens* ; the latter was originally described in 2001 as a species isolated from purulent joint and lung infections in domestic animals, including calves, sheep, and pigs. 5 The two species share as little as 0.5% nucleotide difference on 16S rRNA sequencing, making species-level differentiation challenging without additional molecular methods such as sodA PCR or whole-genome sequencing. 3 6 The dual identification reported in this case ( *G. sanguinis/G. sulfidifaciens* ) reflects this taxonomic difficulty and raises the question of whether some human *Globicatella* infections attributed to *G. sanguinis* may in fact be caused by *G. sulfidifaciens* , or vice versa.

Among the 37 known cases reviewed by Miller et al, 89% occurred at the extremes of age (<5 or >65 years); however, the specific number of neonatal cases is not disaggregated in the published literature. Reported clinical syndromes include bacteremia, meningitis, urinary tract infections, soft tissue infections, osteoarticular infections, and endophthalmitis. 1 3 4 Epidemiology and routes of transmission remain poorly understood; *G. sanguinis* may colonize skin or mucosal surfaces, and human carriage has been demonstrated in groin and rectal specimens, but the reservoir, mode of vertical or horizontal transmission, and risk factors for invasive disease have not been systematically characterized. 1 3 Based on our review of the available case reports, neonatal *Globicatella* bacteremia has been described only in isolated reports, and the present case represents one of the very few documented neonatal infections.

The clinical presentation of this neonate, that is, respiratory distress, lethargy, hypotonia, pallor, tachycardia, hypotension, and metabolic acidosis is consistent with the nonspecific clinical signs of neonatal sepsis described in literature. 1 These signs, however, overlap with other causes of neonatal cardiorespiratory compromise, including hypovolemic shock, perinatal asphyxia, persistent pulmonary hypertension of the newborn, and congenital heart disease. 1

Hypovolemic shock secondary to cord avulsion was the most immediately apparent diagnosis given the witnessed event during delivery, and the rapid hematocrit decline confirmed this diagnosis. Umbilical cord avulsion is a rare but recognized complication of delivery, and spontaneous umbilical cord vascular rupture has historically been associated with rapid neonatal demise. 6

Early-onset neonatal sepsis was confirmed by the positive blood culture within 12 hours. The critical teaching point is that a complete sepsis evaluation, including blood cultures and empiric antibiotic initiation, must be performed in any neonate presenting with hemodynamic instability, even when an obvious hemorrhagic etiology is present. 7 The nonspecific nature of neonatal sepsis signs means that sepsis cannot be excluded on clinical grounds alone, and the two conditions may coexist, as demonstrated in this case.

Additional differential diagnoses considered included the following:

Fetomaternal hemorrhage as an alternative mechanism of blood loss; however, Kleihauer–Betke test was not performed, as the clinical picture was adequately explained by the cord avulsion.

Congenital heart disease can present with cyanosis, respiratory distress, and shock in the immediate neonatal period. 3 8 The rapid resolution of respiratory and hemodynamic compromise with volume resuscitation, the absence of persistent hypoxemia or murmur, and normal pulse oximetry screening made structural heart disease unlikely.

Empiric therapy with ampicillin and gentamicin is the standard of care for suspected EOS in neonates and is recommended by multiple guidelines and systematic reviews. 9 This regimen was initiated promptly following the high index of clinical suspicion. Gentamicin was discontinued after isolation of gram-positive cocci in blood culture, negative CSF cultures, clinical improvement, and repeat culture negativity. Culture-directed de-escalation minimizes unnecessary aminoglycoside exposure and its associated risks of nephrotoxicity and ototoxicity while maintaining effective treatment of the identified pathogen. 10 11

The gram-positive isolate was identified as *G. sanguinis/G. sulfidifaciens.* Antimicrobial susceptibility testing done in national reference laboratory returned MIC values for ceftriaxone (≥4 μg/mL), meropenem (1 μg/mL), penicillin (0.25 μg/mL), and vancomycin (0.25 μg/mL), but all results were reported as “no interpretation” because no CLSI guidelines exist for this organism. This represents a rare scenario in modern clinical microbiology in which a pathogen is identified, susceptibility testing is performed, and yet the results cannot be formally interpreted.

Given the rarity of neonatal *Globicatella* infection, the absence of CLSI breakpoints, and the lack of established treatment guidelines, antimicrobial management required multidisciplinary collaboration with pediatric infectious disease specialists. Subsequently, ampicillin monotherapy was continued for a total of 10 days from the first negative blood culture, consistent with recommended treatment durations for culture-positive neonatal bacteremia without meningitis. 12 The patient’s favorable outcome suggests that prompt recognition and management can result in successful treatment despite the rarity of this organism.

## Conclusion

*Globicatella sanguinis/sulfidifaciens* is a rare but clinically significant cause of early-onset neonatal sepsis, with fewer than five neonatal cases reported in world literature. This case illustrates the diagnostic overlap between hemorrhagic shock and neonatal sepsis, reinforcing the necessity of a complete sepsis evaluation in any neonate with hemodynamic instability, regardless of apparent etiology.

A central challenge was the absence of CLSI breakpoints for *Globicatella* species, rendering antimicrobial susceptibility results uninterpretable by standard criteria and necessitating individualized management guided by infectious disease consultation. As MALDI-TOF MS and molecular identification methods become more widely adopted, the true incidence of *Globicatella* infections may prove higher than currently recognized.

Each additional reported case contributes to the collective understanding of this organism’s clinical significance and antimicrobial susceptibility in neonates and may ultimately support the development of standardized treatment guidelines and CLSI breakpoints for this emerging pathogen.
